# Personality trait associations with quality-of-life outcomes following bariatric surgery: a systematic review

**DOI:** 10.1186/s12955-023-02114-0

**Published:** 2023-03-29

**Authors:** Sarah Summerville, Emma Kirwan, Angelina R. Sutin, Donal Fortune, Páraic S. O’Súilleabháin

**Affiliations:** 1 Department of Psychology, University of Limerick, Limerick, Ireland; 2 Health Research Institute, University of Limerick, Limerick, Ireland; 3 College of Medicine, Florida State University, Tallahassee, FL USA

**Keywords:** Bariatric surgery, Personality, Quality of life, Health-related quality of life, Systematic review

## Abstract

**Background:**

Obesity can be a significant challenge to health and quality of life (QoL). Bariatric surgery assists with weight loss and may help improve QoL. However, not all patients benefit from surgery. Personality traits may be related to QoL outcomes after bariatric surgery, but these associations are unclear.

**Purpose:**

This research reviews the published literature on the associations between personality and QoL among post‐operative bariatric patients.

**Method:**

Four databases (CINAHL Complete, Medline with Full Text, APA PsycINFO, and Scopus) were searched from inception until March 2022. Forward searching was conducted using Google Scholar, and backward reference citation searches were also performed.

**Results:**

Five studies met inclusion criteria yielding data from *N* = 441 post-bariatric patients including both pre/post and cross-sectional designs. Higher agreeableness was related to lower overall health-related QoL (HRQol) and gastric HRQol and positively associated with psychological HRQol. Higher emotional stability was positively related to overall HRQol. Higher impulsivity was negatively associated with mental HRQol and was unrelated to physical HRQol. Effects for the remaining traits were either mainly mixed or null.

**Conclusion:**

Personality traits may be associated with HRQol outcomes. However, it is difficult to reliably discern the role of personality traits for HRQol and QoL outcomes given the methodological issues and few published studies. More rigorous research is needed to address these issues and clarify possible associations.

**Supplementary Information:**

The online version contains supplementary material available at 10.1186/s12955-023-02114-0.

## Introduction

Obesity has significant economic, social, and health implications to individuals and families worldwide [49]. Bariatric surgery is the only treatment intervention that has substantial, long‐term weight loss and medical benefits for those with severe obesity [33, 64]. The Bariatric Analysis and Reporting Outcome System (BAROS) outlines three main areas for assessment of ‘successful’ surgery: percentage of excess weight lost, changes in medical conditions, and quality of life (QoL) [16, 53]. QoL is widely accepted as the perception of one’s position in life, relative to culture and value systems, while considering goals, expectations, standards, and concerns [68].

As per The The WHOQOL Group [68], QoL is a complex concept is thought to reflect physical health, psychological state, level of independence, social relationships, personal beliefs, as well as the individual’s relationship with their lived environment. The WHOQOL Group [68] recommends capturing data on the following factors to comprehensively assess QoL: physical health, psychological state, level of independence, social relationships, relationship with the environment, and personal beliefs. A subcomponent of QoL is health-related quality of life (HRQol), which is how well a person functions in their life relative to their perceived wellbeing in physical, mental, and social domains of health [31]. Though distinct, these terms overlap and have been used interchangeably in the literature [36].

Surgical intervention may yield better improvements in QoL compared to other weight loss methods [22, 42], despite QoL often remaining lower than populations without obesity [1]. Of note, greater benefits have been reported for physical HRQol domains compared with mental HRQol [30, 35, 42] following bariatric surgery. Compared with non-surgical groups for example, improvements in mental QoL are not consistently observed from before to after bariatric surgery [66].

A systematic review of reviews [38] highlighted the relationship between weight loss and improved HRQol after bariatric surgery compared with controls. Outcomes varied greatly and some studies did not find any improvement. A range of possibilities for these inconsistencies has been hypothesised, including insufficient data due to dropout, lack of adequate follow-up, the emphasis of HRQol as a secondary outcome, and questionable quality of studies due to limited quality assessments [38]. It has also been hypothesised that additional factors may play a role in HRQol outcomes [38]. Indeed, Hindle and colleagues’ systematic review (2017) evaluated the role of early post-surgical psychosocial and weight-loss determinants of QoL (among other outcomes). This seminal work illustrates the complexity of QoL outcomes following surgery which results from multiple factors, including psychological determinants [32]. Individual differences, particularly personality traits, may be one aspect that contributes to the variability in QoL outcomes following bariatric surgery.

Five Factor Model (FFM) personality traits are defined as dimensions of relatively stable tendencies in thoughts, emotions, and behaviours across time [46]. Several models exist [3, 13, 24, 56] that purport the implications of individual personality differences. According to the FFM, individuals vary across five traits that contribute to behavioural tendencies over time [46]. Such tendencies may culminate in consequential health outcomes, including obesity across the lifespan [65], risk of future disease [67] and all cause-mortality [29, 52]. Growing evidence suggests that personality traits may also be critically important to consider within the context of QoL outcomes across a variety of populations [4, 19, 21, 26, 39, 57, 59, 73]. Yet to date, there is a lack of clarity on the relation between personality traits and QoL outcomes in bariatric populations.

Wimmelmann et al. [76] narrative review suggested that two personality traits (agreeableness and ‘neurotic predisposition’) were among the psychological variables associated with HRQol outcomes following bariatric surgery. However, studies identified in their review were limited and were not identified through systematic review procedures. There was also a lack of specificity on eligibility criteria, and unstandardised ‘composite’ traits were included as well as traits measured through established well-validated scales. Bordignon et al. [7] conducted a systematic review on personality traits and bariatric surgery outcomes, including QoL. Bordignon et al. [7] found that neuroticism had no association with QoL outcomes, in contrast to extensive findings among other health populations [4, 39, 59].

The association between personality traits and QoL outcomes following bariatric surgery remains unclear and warrants further investigation. We sought to examine studies of personality traits and QoL outcomes following bariatric surgery, regardless of quantitative study design. As such, this research systematically reviews and synthesises findings from the published literature to document the associations between personality traits and QoL outcomes among post‐operative bariatric patients.

## Method

### Protocol registration

This systematic review follows the Preferred Reporting Items for Systematic Reviews and Meta-Analysis (PRISMA) guidelines [54] and is registered within the PROSPERO database (registration number: CRD42021249681).

### Eligibility criteria

The search template guiding this review was structured using the PEO template: Population (bariatric population), Exposure (personality traits), and Outcome (QoL).

#### Population

The population of interest was adult bariatric patients (≥ 18 years) who had undergone weight loss surgery (e.g., Roux-en-Y gastric bypass surgery, sleeve gastrectomy, gastric band, biliopancreatic diversion). Studies that reported data from ≥ 1 year post surgery were considered for inclusion. Studies which only reported data on participants prior to bariatric surgery (e.g., bariatric candidates) were excluded. Research on bariatric weight loss interventions or procedures, other than surgical interventions (e.g., pharmacological) were excluded, as well as surgical interventions where weight loss was not the primary function (e.g., aesthetic body contouring surgery). Surgical research on short term, removable bariatric devices (e.g., gastric balloon) was also excluded.

#### Exposure

All conceptualizations of personality traits [13, 17, 24, 27] were considered for inclusion in the current review, where studies reported the association between traits and QoL outcomes.

#### Outcome

Studies that reported QoL or HRQol, measured by multi-item or single item scales were included.

#### Studies

Peer reviewed publications including descriptive or analytical observational studies using either prospective (cohort studies) or retrospective (e.g., case control) data collection approaches were eligible for inclusion. Experimental studies were also eligible for inclusion (e.g., RCT). Articles that met exclusion criteria for the current review included other reviews, meta-analyses, letters to the editor, conference abstracts, or empirical commentaries. Qualitative studies were also excluded as well as research not published in English.

### Information sources

The search was conducted from inception until March 2022, using the following electronic databases: CINAHL Complete, Medline with Full Text, APA PsycINFO through EBSCOhost; and Scopus. Forward (using Google Scholar) and backward reference citation searches were performed to identify additional relevant studies from the final articles. See Table 1 of supplementary material for Google Scholar search results. Search results were documented and saved within EndNote folders throughout the data collection period and email alerts were activated to capture new studies.

### Search strategy

A search strategy was devised in line with Peer Review of Electronic Search Strategies (PRESS) [48], supported by an expert librarian and agreed upon by the review team. The search was executed in accordance with the PRISMA-S checklist [62] to complement the PRISMA Statement [54]. A combination of key words with MESH/subject headings/ thesaurus terms were utilised within each search engine. Search terms were developed across three concepts of ‘bariatric surgery’ ‘AND’ ‘trait’ ‘AND’ ‘quality of life’ and applied to each database. Surgical search terms were informed by prior works from Hindle et al. [32] and Sheets et al. [63]. See search strings in supplementary material, Table 2. To prevent missing unindexed articles, each search was performed in all text [TX]. Appropriate Boolean operators were applied in these instances. No cut-off date was applied to article publication and only peer-reviewed articles, published in English, within academic journals were included.

### Selection process

Search results from each database were exported into EndNote. After duplicates were removed, 100% of the articles were independently blind screened (titles and abstracts) by two reviewers (SS and EK) using ‘Rayyan’ software, according to the eligibility criteria. Consensus was reached on these results and 100% of full-text articles were then independently blind-screened. Disagreement on one occasion was resolved by a third reviewer (PO). Cohen’s Kappa coefficient ranged from 0.67 to 0.92 during the screening stages, indicating high inter-rater reliability between reviewers [14].

### Risk of bias

The internal validity of included studies was assessed using the US National Heart Lung and Blood Institute (NHLBI) quality appraisal tools, developed by the National Institutes of Health [51]. ‘Pre/post studies with no control group’ (PPSC) and ‘Observational cohort and cross-sectional studies’ (OCCSS) were deemed appropriate for the current research [44]. ‘Item 11’ within the PPSC was replaced with ‘item 14’ from the OCCSS, to account for confounding variables. The NHLBI tools included items to evaluate potential flaws promoting risk of bias (e.g., participant selection, attrition, confounders, study power, strength of causality etc.). These tools assist reviewers to focus on central concepts for critical appraisal of internal validity. Responses were “yes” (Y), “no” (N), “not applicable” (NA), “not reported” (NR) or “cannot determine” (CD). These tools were not designed to provide a list of factors comprising a numeric score [51]. Reviewers therefore considered the risk of bias presented by each respective flaw, relative to every study. This data was used to guide the overall quality rating as either “good,” “fair,” or “poor”. One author (SS) independently evaluated tool items for all studies and a second reviewer (EK) reviewed 60% of these.

### Data extraction process and synthesis

Study characteristics tabulated to describe relevant study features are reported in Tables 1 and 2 (main effects were reported separately– see Table 3 in supplementary material). To avoid data extraction errors, spot checks were conducted by a second reviewer (EK) for 40% of articles. No errors were identified from these checks. Authors from three studies were contacted about missing data and reminder emails were sent for follow-up. Two authors responded with feedback. According to best practice guidelines for meta-analysis [20], it was not possible to employ quantitative analyses within this review. Small sample sizes, heterogeneity of measurement tools, variability of analyses, and differences in confounding variables indicated that narrative synthesis would best serve the data. Narrative ‘Synthesis Without Meta-analysis’ (SWiM) employing textual description of results was used. To provide transparency in narrative reporting, ‘SWiM’ guidelines were applied where possible in accordance with recommendations by Popay et al. [61] and Campbell et al. [9].

**Table 1:** Study Characteristics

Author	Design	*N*	Sex	Mean age	Country	Follow up	Operation	Personality trait assessed	Relevant outcome variable
Caltabiano [8]	Cross-sectional	127	Female (only females recruited) (100%)	45	Online	N/A	• 73.9% vertical sleeve • 18.5% gastric bypass • 7.6% gastric band	• Extraversion, • Conscientiousness, Agreeableness, • Intellect/Imagination, • Emotional stability	HRQol: Total Obesity related wellbeing, relevance of symptoms on QoL, occurrence of symptoms on QoL
Pereira et al. [58]	Prospective Pre/post	90	*n* = 79 female (87.78%)	45.14	Portugal	1 year	• Gastric bypass (85.6%) • Gastric sleeve (14.4%)	• Impulsivity	HRQol mental QoL, physical QoL
Canetti et al. [11]	Prospective Pre/post	51 (bariatric surgery) 51 (diet)	*n* = 44 female (86.27) *n* = 33 female (64.71%)	34.2	Israel	1 year	• Silastic ring vertical banded gastroplasty (*n* = 44) • Laparoscopic adjustable gastric band (*n* = 7)	• Neuroticism	HRQol
Lee et al. [41]	Pre/ post prospective study	61	*n* = 49 female (80.33%)	33.3	China	1 year	• Laparoscopic sleeve gastrectomy	• Competence • Agreeableness • Extraversion, • Open-mindedness	HRQol: Total Gastrointestinal QoL (GIQoL), social domain, psychological domain, psychical domain
Van Hout et al. [69]	Pre/post prospective study	112	*n* = 98 female (87.5%)	38.8	Netherlands	2 years	• Laparoscopic vertical band gastroplasty	• Neurotic lability-somatic symptoms	HRQol

**Table 2:** Summary of results and main effects

Author	Trait measurement tool	QoL measurement tool	Analysis performed	Control variables	Main effects
Caltabiano [8]	International Personality Item Pool (IPIP)	Obesity-Related Well-being scale (ORWELL 97)	Pearson correlation Hierarchical regression	- BMI	**Associations between personality traits and Orwell 97 domains:** Extraversion and total Orwell 97: (*r* = -.19, *p* < .05) Conscientiousness and total Orwell 97: (*r* = -.21, *p* < .05) Emotional stability and total Orwell 97: (*r* =—.47, *p* < .01) All other traits, not significant **Personality trait impact upon total Orwell 97:** Agreeableness and Total Orwell 97: (β = 0 .27, *p* < .01) Emotional stability and Total Orwell 97: (β =—.43, *p* = .001) All other traits, not significant **Personality trait impact upon Orwell 97 symptom occurrence** Agreeableness and Orwell 97 symptom occurrence: (β = .29, *p* < .01) Emotional stability and Orwell 97 symptom occurrence: (β—.47, *p* = .001) All other traits not significant **Personality trait impact upon Orwell 97 symptom relevance** Agreeableness and Orwell 97 symptom relevance: (β = .25, *p* < .05) Emotional stability and Orwell 97 symptom relevance: (β = -.36, *p* = .001) All other personality traits not significant
Pereira et al., [58]	Barratt Impulsivity Scale (BIS)	Short Form Health Survey–SF 36	Pearson correlation Hierarchical regression Moderation analysis	- Professional status	Negative association between impulsivity and mental QoL: (*r* = -.62, *p* < .001) Negative association between impulsivity and physical QoL: (*r* =—42, *p* < .001) Impulsiveness negatively related to mental QoL: (β = -.33, *t* = -3.88, *p* < .001) Negative impact of impulsivity upon mental QoL and physical QoL is significantly moderated by post-surgery spirituality: (*t* = − 2.57, *p* = .01) (*t* = − 2.04, *p* = .04)
Canetti et al., [11]	NEO- Personality Inventory Revised (neuroticism scale) NEO-PI-N)	Medical Outcome Survey Short Form – 36 (MOS SF-36)	Corelation analysis	Correlation analysis: Initial level of the outcome variables: weight loss, HRQol, mental health, psychological distress and wellbeing	Partial correlation coefficients Neuroticism and QoL: (*r* = -0.03, *p* > .05 (no significant association detected)
Lee et al., [41]	Chinese Personality Assessment Inventory (CPAI)	Gastrointestinal Quality-of-Life Index	Students t-test / Wilcoxon test	-	**Effect sizes and specific significance values were not reported** Agreeableness significantly negatively associated with in ‘gastrointestinal’ domain of QoL: (*p* ≤ .05) Agreeableness significantly associated with ‘emotional’ QoL: (*p* ≤ .05) Extraversion significantly positively associated with social functioning of QoL: *p* ≤ .05 Extraversion significantly positively impacted total change in GIQoL: (*p* ≤ .05) No relationship detected for other personality traits (competence and open-mindedness) and QoL domains (GI, social, psychological, physical, total)
van Hout, et al., [69]	Amsterdam Biographical Questionnaire (ABQ)	Rand-36 Short Form Health Survey (Rand SF-36)	Hierarchical regression	Age, gender, preoperative BMI	Neurotic lability somatic symptoms No significant relationship, not described in reported model

## Results

**Fig. 1:**
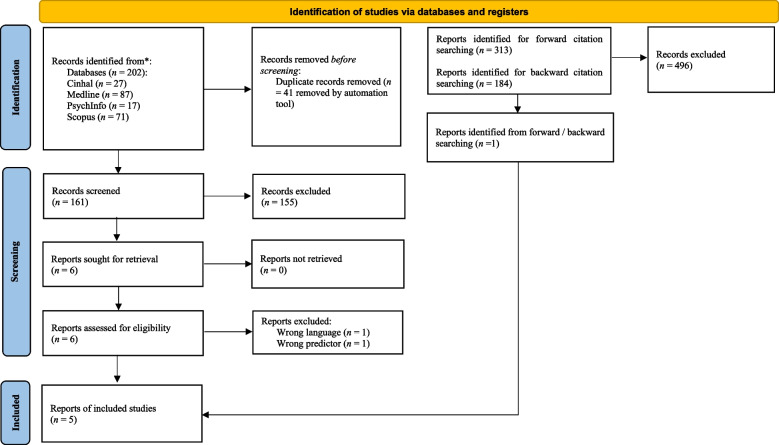
Summary PRISMA flow chart ([54]

The database search yielded a total of 161 records after duplicates were removed. A further 155 records were removed from screening titles and abstracts. Full texts were retrieved for six articles which appeared to meet inclusion criteria. Two studies were excluded: one because the authors did not assess the relationship between personality and QoL [10]; the other study was not reported in English [2]. Forward and backward citation searches were conducted for the four articles that met inclusion criteria and resulted in one additional study [58]. A total of five articles met the inclusion criteria. Figure 1 illustrates the PRISMA flow diagram.

### Study characteristics

Studies included within this review were published between 2009 – 2021 and provide data on a total of *N* = 441 post-bariatric patients. Most were female; due to some non-reported data from loss to follow-up, the exact number of female participants is unclear. Bariatric samples ranged from *n* = 51 to *n* = 127. Studies were conducted across multiple countries including Portugal [58], the Netherlands [69] China [41], and Israel [11]. Research by Caltabiano [8] was conducted online and participant demographics were not reported. Attrition was not applicable to Caltabiano [8] due to the cross-sectional design. Lee et al. [41] reported no dropouts within their sample, whereas Canetti et al. [11] assessed and reported no significant differences between initial measures from completers and non-completers. Neither Pereira et al. [58] or Van Hout et al. [69] described statistical investigation of loss to follow-up.

Of the five studies included in this review, participants underwent one of four surgical techniques. Two studies [8, 11] reported methods using the adjustable gastric band method. Two studies [11, 69]) reported participants who underwent laparoscopic vertical band gastroplasty. Three studies [8, 41, 58] also reported data from participants who underwent the gastric sleeve technique. Finally, participants within two articles [8, 58] received the gastric bypass method. Study characteristics are summarised in Table 1.

#### Study designs

One study [8] was cross-sectional, and reported Pearson correlation analyses and hierarchical regression analyses to assess the data. Four studies [41, 58, 69] had a prospective, pre/post research designs to assess participants at two time-points (pre-surgery and post-surgery). Van Hout et al. [69] also reported hierarchical multiple regression analyses. Canetti et al. [11] used a pre/post design and compared group outcomes between individuals who underwent bariatric surgery and controls (others in a weight-loss diet programme). To assess outcomes, they used correlation analyses and subsequently applied confirmatory structural equation modelling for appropriate variables. Pereira et al. [58] assessed participants before and after surgery, applying correlational analyses, hierarchical regression analyses and moderation analyses, to test for effects. Lee et al. [41] reported t-tests to summarise their results.

#### Personality inventories

Pereira et al. [58] used the Portuguese version [70] of the Barratt Impulsivity Scale (BIS) [55] to assess levels of trait impulsivity. Higher scores on this scale indicate greater impulsiveness. Caltabiano [8] used the International Personality Item Pool (IPIP) [27], which measures the five FFM traits: extraversion, agreeableness, conscientiousness, emotional stability (neuroticism), and intellect. Canetti et al. [11] measured neuroticism using the NEO- Personality Inventory Revised (NEO-PI-N). Canetti and colleagues (2009) also assessed a “neurotic predisposition” (NP). This concept, however was not assessed in the present review because it is not a validated personality trait construct. Lee et al. [41] assessed personality traits using the Chinese Personality Assessment Inventory (CPAI) [12], which measures factors from the ‘Big Seven’ model of Chinese indigenous personality [71]: agreeableness, competence, contentedness, extraversion, industriousness, open-mindedness, and other-orientation. Lee et al. [41] only reported findings for agreeableness, competence, extraversion, and open-mindedness. Finally, the Amsterdam Biographical Questionnaire (ABQ) [75] was used by Van Hout et al. [69] to assess neurotic lability-somatic symptoms. The ABQ is a personality inventory with four scales: neuroticism (psycho-neurotic complaints), neurosomatism (functional somatic complaints), extraversion, and social conformity or lie.

#### HRQol scales

One study [8] measured HRQol using the Obesity Related Well-Being questionnaire (ORWELL-97). The ORWELL-97 provides an overall HRQol score and two subscale scores: symptom occurrence (occurrence and severity of obesity-related symptoms e.g. “Does your weight interfere with your social activities?”) and relevance of symptoms (resultant subjective impairment in life e.g. “Is it important for you to spend your free time with friends?”) [45]. Higher scores indicate lower HRQol [45]. Another study [41] employed the Gastrointestinal Quality of Life Inventory (GQLI) to assess HRQol outcomes, yielding an overall HRQol score and four subscales: gastrointestinal symptoms, emotions (psychological), physical functions, and social functions. Higher scores on the GQLI indicate better HRQol [23]. The 36-Item Short Form Survey (SF-36) was used in two studies [58, 69] to assess HRQol. The SF-36 yields an overall score and two subscale scores: mental HRQol and physical HRQol. Higher scores indicate a more favourable health state [72].

### Risk of bias results

Reviewers reached 100% agreement regarding quality assessment for each study. Results from the risk of bias assessment rated three articles [8, 58, 69] as “good”, indicating low risk of bias. One article [11] was rated as “fair”, indicating medium risk of bias. Canetti et al. [11] indicated their findings to be preliminary based upon an underpowered sample. Despite management of covariates within the analyses, additional uncontrolled variables (weight loss) were suspected to have impacted results. Another article [41] was rated as “poor”, indicating high risk of bias. Major concerns were the lack of covariates, limited eligibility criteria, and statistical under-reporting that challenged the integrity of the results, according to reviewers. Studies included in this review did not control for differences between surgical techniques. As such, in the synthesis below, it is important to evaluate the results from this study with caution given its poor-quality rating. Overall, the internal validity based upon risk of bias for the included studies ranges from poor to good. Further risk of bias results are reported in Additional file 4; Table 4 I – V, Additional file 5; Tables 5 and 6.

### Synthesis of quantitative evidence

#### Neuroticism

The association between neuroticism and HRQol outcomes varied across studies. Canetti et al. [11] tested for an association between neuroticism, measured by the NEO-PI-N scale, and HRQol, measured by the SF-36. The correlation was not significant. Van Hout et al. [69] did not report a significant association between neurotic lability-symptoms measured by the ABQ and physical HRQol according to the SF-36. Caltabiano [8] used a cross-sectional design to investigate the association between neuroticism measured by the IPIP [28] and HRQol according to the ORWEL-97, among female participants after bariatric surgery. Pearson correlations indicated a significant association between emotional stability (inverse of neuroticism) and overall HRQol, symptom occurrence, and symptom relevance. Subsequent hierarchical regression analysis revealed a medium-sized, positive association for emotional stability with overall HRQol. A medium-sized negative association between emotional stability and symptom occurrence and symptom relevance was also reported.

#### Impulsivity, extraversion, and openness

Pereira et al. [58] investigated the association between impulsivity as measured by the BIS [55] and post-surgical mental and physical HRQol, using the SF-36. Impulsivity was significantly associated with mental QoL [58]. No relationship between impulsiveness and physical HRQol was identified [58]. Results were mixed for extraversion. Lee et al. [41] found that extraversion was positively associated with overall HRQol and with social HRQol, whereas Caltabiano [8] found no significant associations between extraversion and HRQol. There was no association between openness and HRQol outcomes. Caltabiano [8] found that openness (measured as intellect imagination) was not significantly associated with HRQol (total, symptom relevance, or symptom occurrence). Open-mindedness, a concept within the Chinese ‘Big Seven’ model of personality which is somewhat similar to FFM openness [71] was also unrelated to all HRQol outcomes [41].

#### Agreeableness and conscientiousness

Caltabiano [8] found a medium-sized negative association between agreeableness and overall HRQol, with a medium positive association with symptom relevance and symptom occurrence. Lee et al. [41] identified similar associations with agreeableness as part of the Chinese ‘Big Seven’ personality model. Agreeableness was negatively associated with gastrointestinal symptoms (suggesting more gastrointestinal complaints) but was positively associated with psychological HRQol, suggesting higher psychological HRQol. Agreeableness was unrelated to physical HRQol and overall HRQol [41]. Conscientiousness was unrelated to HRQol [8]. Competence, a factor somewhat similar to conscientiousness within the Chinese ‘Big Seven’ Model of personality (differs from FFM competence) [37], was unrelated to HRQol outcomes [41]. Other traits within the Chinese Big Seven (industriousness, other-orientation, and contentedness) were not reported by Lee et al. [41].

## Discussion

This review applied a rigorous systematic approach to appraise and synthesise evidence for an association between personality traits and QoL outcomes among post-surgical bariatric patients. This was the first systematic review to report evidence from all quantitative designs on studies that measured personality from a trait perspective. Overall, neuroticism appears to be associated with worse HRQol, whereas extraversion was found to be associated with higher HRQol, although null associations were also found for these two traits. The association was most mixed for agreeableness, with both positive and negative associations depending on the HRQol domain. Openness and conscientiousness were unrelated to HRQol. Few published studies, however examined the associations between personality and HRQol outcomes in this population.

Findings surrounding possible associations with neuroticism were mixed. The negative association between neuroticism and QoL outcomes is well established among other populations such as general health [57, 59], chronic disease [4], and breast cancer [39]. Two studies identified in this review found no significant association for neuroticism [11, 69]. Both studies had small sample sizes that may have limited power to detect significant associations. Conversely, Caltabiano [8], with a substantially larger sample size did identify significant associations. Therefore, these findings may have been due to sampling issues, rather than a unique nuance within the bariatric population. It is well established that those high in neuroticism (low emotional stability) are more predisposed to stress, self-consciousness, and impulsivity than those lower in neuroticism (high emotional stability) [17]. As such, individuals lower in emotional stability may perceive their post-surgical aliments as highly threatening and ruminate over these in the potential absence of robust coping mechanisms.

Impulsivity is typically viewed as a subcomponent of neuroticism within the FFM [17, 18, 34] and is the reactive predisposition to act without planning or considering the consequences [50]. Impulsivity may be associated with worse psychological HRQol outcomes [58]. Links between mental health challenges and neuroticism are well established [43], therefore findings from Pereira et al. [58] are consistent with research expectations. Though personality traits are heterogeneous among bariatric patients [25], evidence suggests that impulsivity is associated with weight status [6], and thus is likely to present in greater proportions among bariatric populations, highlighting potential challenges for surgical patients.

Agreeableness may have unique implications for HRQol due to socially adaptive features of this trait, but studies are sparse, and results are inconsistent. This prosocial trait is defined as being trustful and forgiving, straightforward, modest, compliant, tenderminded, and altruistic [18, 47]. Therefore, improvements to psychological HRQol among bariatric patients who score higher in agreeableness [41] are unsurprising and remain consistent with other psychological research [40]. Remarkably, higher agreeableness was also related to worse outcomes in physical HRQol domains. Similarly, to neuroticism, these findings need to be replicated to determine whether this discrepancy is due to chance or whether there is a substance difference across HRQol domains. No associations were reported for conscientiousness and openness.

Several challenges limit the literature on personality traits and QoL outcomes. First, most studies are observational and use linear models such as cross-sectional and pre/post designs. Although useful, more samples are needed that measure QoL at multiple points over time to reliably detect change. Second, there is a lack of control variables in many of the included studies that presents significant challenges to reliably interpreting the data. More careful consideration of covariates can help to better identify trait effects. Third, there were recruitment issues, and some studies were predominantly female. Despite the higher global incidence of females using bariatric surgery [74], the under representation of males may impact the generalisability of results to the wider bariatric population. Future research should increase equity of access for males in bariatric research. Fourth, the heterogeneity of traits assessed within this literature needs to be addressed in future research. Two studies only examined a single personality trait. Future research needs to examine the potential relevance of each of the broad personality traits from the Five Factor Model of Personality and their underlying facets to provide a more comprehensive assessment.

### Strengths and limitations

Two major strengths of the present study are the comprehensive search of the literature across multiple databases and the rigorous methodology employed. Conversely, the main limitation of this systematic review is the few studies included, due to the paucity of published research. This limits the inferences and recommendations possible for research and practice. Owing to the infancy of such investigation, this review includes research impacted by bias which further impedes comparisons. Despite this inclusion, research with a poor-quality rating helped to clarify what future research in the area needs to address. Additionally, of the three relevant studies that reported dropouts [11, 58, 69], comparisons between included participants and those lost to follow-up were described in only one study [11]. Attrition is common and problematic in bariatric populations [15, 60]. Therefore, methods to comprehensively report patient data following surgery are necessary to promote meaningful understanding in the area. Furthermore, despite including validated HRQol scales, heterogeneity among these in the current review constrained interpretation and encouraged greater emphasis on health rather than QoL. For example, Lee et al. [41] employed the GIQLI. However, many of the GIQLI items measure gastrointestinal symptoms and physical HRQol rather than broader components of HRQol. This impacts comparability of results and requires caution when interpreting QoL outcomes across the bariatric population.

Additionally, Grading of Recommendations Assessment, Development and Evaluation (GRADE) analysis [5] was not conducted for studies within the current review as per SWiM guidelines [9], due to the low levels of evidence associated with the included observational study designs. Furthermore, papers were limited to those published in English which favours researchers’ bias towards their native language and constrains the breadth of evidence available. Finally, due to the unique yet overlapping dimensions which HRQol and QoL present, it is preferable to report these separately. However, this was not possible in the current review as research investigating personality traits and multifaceted QoL outcomes for post-surgical bariatric patients has not yet been conducted. This highlights a significant gap in the research. Instead, all included studies explored the associations between personality traits and HRQol, which can be understood as only one component of overall QoL. Research focused on HRQol alone, limits the universality required to inform overall adjustment to life after bariatric surgery. To address this, future research would benefit from exploring associations between personality traits and the multifactorial components of QoL.

## Conclusion

Overall, findings from the current review offer emerging evidence that personality traits may impact HRQol outcomes following bariatric surgery. However, considering both the methodological issues and paucity of research in the area, it is difficult to reliably discern the role and mechanisms which personality traits play in HRQol and QoL outcomes. This finding is compounded by the lack of study replication over time. Research in the area remains concerned with health and HRQol outcomes rather than broader, multifaceted markers to adjustment in life after bariatric surgery. To this end, it is surprising that research investigating the role of personality traits and QoL outcomes following bariatric surgery does not exist. Therefore, more research is needed to address the issues identified in the current review, and to clarify the association between personality traits and QoL outcomes among people who undergo bariatric surgery.

## Supplementary Information

**Additional file 1: Table 1.** Supplementary material A. Results from ‘forward’, and ‘backward’ citation search.

**Additional file 2: Table 2.** Search strings.

**Additional file 3: Table 3.** Summary of results and main effects.

**Additional file 4: Table 4.** I – V. Risk of bias results data.

**Additional file 5: Table 5.** Quality assessment for observational cohort and cross-sectional studies. **Table 6.** Quality assessment for pre/post studies with no control.

## Data Availability

All review data are available within the manuscript and supplementary materials.

## Abbreviations

BMI: Body mass index
PRISMA: Preferred Reporting Items for Systematic reviews and Meta-Analyses
NHLBI: (US) National Heart Lung and Blood Institute
HRQol: Health-Related Quality of Life
SWiM: Synthesis without meta-analysis
QoL: Quality of life
