# Supplementary material for: Personality trait associations with quality-of-life outcomes following bariatric surgery: a systematic review
Source: Health Qual Life Outcomes. 2023 Mar 29;21:32. doi: 10.1186/s12955-023-02114-0 (PMC10061792; doi:10.1186/s12955-023-02114-0)
Supplement: Supplementary file 1 — Additional file 1: Table 1. Supplementary material A. Results from ‘forward’, and ‘backward’ citation search. [file 12955_2023_2114_MOESM1_ESM.docx]

**Additional file 1.**

**Table 1. Supplementary material A. Results from Google Scholar ‘forward’, and ‘backward’ citation search**

| Search last conducted 09/07/2021 | | | | |
| --- | --- | --- | --- | --- |
| Article | Backward | | Forward | |
|  | Retrieved | Relevant | Retrieved | Relevant |
| Caltabiano, M. L. (2021). Personality, weight loss and obesity-related well-being post-bariatric surgery. *Eating and Weight Disorders-Studies on Anorexia, Bulimia and Obesity*, 1-8. | 46 | 0 | 0 | 0 |
| Lee, Y. C., Lee, C. K., Liew, P. L., Lin, Y. C., & Lee, W. J. (2011). Evaluation of quality of life and impact of personality in Chinese obese patients following laparoscopic sleeve gastrectomy. *Hepato-gastroenterology*, *58*(109), 1248-1251. | 21 | 0 | 4 | 0 |
| Canetti, L., Berry, E. M., & Elizur, Y. (2009). Psychosocial predictors of weight loss and psychological adjustment following bariatric surgery and a weight‐loss program: The mediating role of emotional eating. International Journal of Eating Disorders, 42(2), 109-117. | 57 | 0 | 225 | 1 |
| Pereira, M. G., Faria, S., & Lopes, H. (2019). Quality of Life One Year After Bariatric Surgery: the Moderator Role of Spirituality. *Obesity surgery*, *29*(4), 1207-1215. |  |  |  | Include |
| Wimmelmann, C. L., Smith, E., Lund, M. T., Hansen, M., Dela, F., & Mortensen, E. L. (2015). The psychological profile of bariatric patients with and without type 2 diabetes: baseline results of the longitudinal GASMITO-PSYC study. *Surgery for Obesity and Related Diseases*, *11*(2), 412-418. |  |  |  | Compared groups, did not test personality* on QOL  Exclude |
| Sockalingam, S., Hawa, R., Wnuk, S., Santiago, V., Kowgier, M., Jackson, T., ... & Cassin, S. (2017). Psychosocial predictors of quality of life and weight loss two years after bariatric surgery: Results from the Toronto Bari-PSYCH study. *General hospital psychiatry*, *47*, 7-13. |  |  |  | *Incorrect predictors*  Exclude |
| van Hout, G. C., Hagendoren, C. A., Verschure, S. K., & van Heck, G. L. (2009). Psychosocial predictors of success after vertical banded gastroplasty. Obesity surgery, 19(6), 701-707. |  |  | 80 | 0 |
| Caltabiano, M. L. (2021). Personality, weight loss and obesity-related well-being post-bariatric surgery. *Eating and Weight Disorders-Studies on Anorexia, Bulimia and Obesity*, 1-8. |  |  |  | *Already included*  Exclude |
| Rodríguez-Hurtado, J., Ferrer-Márquez, M., Fontalba-Navas, A., García-Torrecillas, J. M., & Olvera-Porcel, M. C. (2017). Influence of psychological variables in morbidly obese patients undergoing bariatric surgery after 24 months of evolution. *Cirugía Española (English Edition)*, *95*(7), 378-384. |  |  |  | QoL not assessed and Pathological personality  Exclude |
| Lier, H. Ø., Biringer, E., Hove, O., Stubhaug, B., & Tangen, T. (2011). Quality of life among patients undergoing bariatric surgery: associations with mental health-A 1 year follow-up study of bariatric surgery patients. *Health and quality of life outcomes*, *9*(1), 1-10. |  |  |  | Personality disorder  Exclude |
| Miras, A. D., Al-Najim, W., Jackson, S. N., McGirr, J., Cotter, L., Tharakan, G., ... & Scholtz, S. (2015). Psychological characteristics, eating behavior, and quality of life assessment of obese patients undergoing weight loss interventions. *Scandinavian Journal of Surgery*, *104*(1), 10-17. |  |  |  | Outcome weight-loss  Exclude |
| Peterhänsel, C., Nagl, M., Wagner, B., Dietrich, A., & Kersting, A. (2017). Predictors of changes in health-related quality of life 6 and 12 months after a bariatric procedure. *Obesity surgery*, *27*(8), 2120-2128. |  |  |  | Personality not assessed  Exclude |
| Pereira, M. G., Faria, S., & Lopes, H. (2019). Quality of Life One Year After Bariatric Surgery: the Moderator Role of Spirituality. *Obesity surgery*, *29*(4), 1207-1215. | 60 | 0 | 4 | 0 |
|  |  |  |  |  |
| Total | 184 |  | 313 | 1 |
