# Supplementary material for: Personality trait associations with quality-of-life outcomes following bariatric surgery: a systematic review
Source: Health Qual Life Outcomes. 2023 Mar 29;21:32. doi: 10.1186/s12955-023-02114-0 (PMC10061792; doi:10.1186/s12955-023-02114-0)
Supplement: Supplementary file 2 — Additional file 2: Table 2. Search strings. [file 12955_2023_2114_MOESM2_ESM.docx]

**Additional file 2.**

**Table 2. Search strings.**

***Example systematic search terms (EBSCOHOST, Medline Full Text)***

| **Search number** | **Search term** |
| --- | --- |
| **S1** | (MH "Bariatric Surgery+") |
|  |  |
| **S2** | TI ( "bariatric surgery" OR "weight loss surgery" OR "metabolic surgery" OR "gastric bypass" OR "gastric band" OR "gastric sleeve" OR "gastroplasty" OR "sleeve gastrectomy" OR "biliopancreatic diversion" OR "duodenal switch" OR "roux-en-y" OR GBP OR RYGB ) OR AB ( "bariatric surgery" OR "weight loss surgery" OR "metabolic surgery" OR "gastric bypass" OR "gastric band" OR "gastric sleeve" OR "gastroplasty" OR "sleeve gastrectomy" OR "biliopancreatic diversion" OR "duodenal switch" OR "roux-en-y" OR GBP OR RYGB ) |
| **S3** | TX Personality or trait or facet or temperament |
| **S4** | TX “Quality of life” or QoL |
|  |  |
