# Supplementary material for: Personality trait associations with quality-of-life outcomes following bariatric surgery: a systematic review
Source: Health Qual Life Outcomes. 2023 Mar 29;21:32. doi: 10.1186/s12955-023-02114-0 (PMC10061792; doi:10.1186/s12955-023-02114-0)
Supplement: Supplementary file 3 — Additional file 3: Table 3. Summary of results and main effects. [file 12955_2023_2114_MOESM3_ESM.docx]

| **Table 3. Summary of results and main effects** | | | | | |
| --- | --- | --- | --- | --- | --- |
| **Author** | **Trait measurement tool** | **QoL**  **measurement tool** | **Analysis performed** | **Control variables** | **Main effects** |
|  |  |  |  |  |  |
| Caltabiano (2021) | International Personality Item Pool  (IPIP) | Obesity-Related Well-being scale (ORWELL 97) | Pearson correlation  Hierarchical regression | -  BMI | **Associations between personality traits and Orwell 97 domains:**  Extraversion and total Orwell 97: (*r =* -.19, *p* < .05)  Conscientiousness and total Orwell 97: (*r =* -.21, *p* < .05)  Emotional stability and total Orwell 97: (*r =* - .47, *p* < .01)  All other traits, not significant  **Personality trait impact upon total Orwell 97:**  Agreeableness and Total Orwell 97: (β = 0 .27, *p* <.01)  Emotional stability and Total Orwell 97: (β = - .43, *p* = .001)  All other traits, not significant  **Personality trait impact upon Orwell 97 symptom occurrence**  Agreeableness and Orwell 97 symptom occurrence: (β = .29, *p* < .01)  Emotional stability and Orwell 97 symptom occurrence: (β - .47, *p* = .001)  All other traits not significant  **Personality trait impact upon Orwell 97 symptom relevance**  Agreeableness and Orwell 97 symptom relevance: (β = .25, *p* < .05)  Emotional stability and Orwell 97 symptom relevance: (β = -.36, *p* = .001)  All other personality traits not significant |
| Pereira et al., (2019) | Barratt Impulsivity Scale (BIS) | Short Form Health Survey–SF 36 | Pearson correlation  Hierarchical regression  Moderation analysis | -  Professional status | Negative association between impulsivity and mental QoL: (*r* = -.62, *p* < .001)  Negative association between impulsivity and physical QoL: (*r* = - 42, *p* < .001)  Impulsiveness negatively related to mental QoL: (β = -.33, *t* = -3.88, *p* < .001)  Negative impact of impulsivity upon mental QoL and physical QoL is significantly moderated by post-surgery spirituality:  (*t* = − 2.57, *p* = .01)  (*t* = − 2.04, *p* = .04) |
| Canetti et al., (2009) | NEO- Personality Inventory Revised (neuroticism scale)  NEO-PI-N) | Medical Outcome Survey Short Form – 36 (MOS SF-36) | Corelation analysis | Correlation analysis:  Initial level of the outcome variables: weight loss, HR-QoL, mental health, psychological distress and wellbeing. | Partial correlation coefficients  Neuroticism and QoL: ( *r* = -0.03, *p* > .05 (no significant association detected) |
| Lee et al., (2011) | Chinese Personality Assessment Inventory (CPAI) | Gastrointestinal Quality-of-Life Index | Students t-test / Wilcoxon test | - | **Effect sizes and specific significance values were not reported.**  Agreeableness significantly negatively associated with in ‘gastrointestinal’ domain of QoL: (*p* ≤ .05)  Agreeableness significantly associated with ‘emotional’ QoL: (*p* ≤ .05).  Extraversion significantly positively associated with social functioning of QoL: *p* ≤ .05  Extraversion significantly positively impacted total change in GIQoL: (*p* ≤ .05)  No relationship detected for other personality traits (competence and open-mindedness) and QoL domains (GI, social, psychological, physical, total) |
| van Hout, et al., (2009). | Amsterdam Biographical Questionnaire  (ABQ) | Rand-36 Short Form Health Survey (Rand SF-36) | Hierarchical regression | Age, gender, preoperative BMI | Neurotic lability somatic symptoms  No significant relationship, not described in reported model |
