# Supplementary material for: Personality trait associations with quality-of-life outcomes following bariatric surgery: a systematic review
Source: Health Qual Life Outcomes. 2023 Mar 29;21:32. doi: 10.1186/s12955-023-02114-0 (PMC10061792; doi:10.1186/s12955-023-02114-0)
Supplement: Supplementary file 4 — Additional file 4: Table 4. I – V. Risk of bias results data. [file 12955_2023_2114_MOESM4_ESM.docx]

**Additional file 4.**

**Table 4: I – V****. Risk of bias results data.**

| **I. Caltabiano (2021)** | | | |
| --- | --- | --- | --- |
| **Quality Assessment Tool for Observational Cohort and Cross-Sectional Studies** | **Y** | **N** | **Other**  **(CD, NR, NA)*** |
| 1. Was the research question or objective in this paper clearly stated?  *“The aim of the study was to examine the associations between personality, weight loss and obesity-related wellbeing post-bariatric surgery”* | Y |  |  |
| 2. Was the study population clearly specified and defined?  *“A sample of 127 females who had received bariatric surgery answered an online survey ….”.* Defined time since surgery not reported for sample. | Y |  |  |
| 3. Was the participation rate of eligible persons at least 50%? |  |  | CD |
| 4. Were all the subjects selected or recruited from the same or similar populations (including the same time period)? Were inclusion and exclusion criteria for being in the study prespecified and applied uniformly to all participants?  Online survey - time period not outlined.  Bariatric surgery: vertical sleeve gastric bypass, and gastric band. Two respondents had the intragastric balloon and were removed. | Y |  |  |
| 5. Was a sample size justification, power description, or variance and effect estimates provided?  *“For multiple hierarchical regression to be powered, a sample size of 98 was required (N >or equal to 50+8 m, where m is the number of predictor variables p. 117) …. There were six predictor variables, so our sample of 125 participants was powered to detect significant effects.”* | Y |  |  |
| 6. For the analyses in this paper, were the exposure(s) of interest measured prior to the outcome(s) being measured?  Cross-sectional design, therefore predictors and outcomes were measured at same time. |  | N |  |
| 7. Was the timeframe sufficient so that one could reasonably expect to see an association between exposure and outcome if it existed? |  |  | N/A |
| 8. For exposures that can vary in amount or level, did the study examine different levels of the exposure as related to the outcome (e.g., categories of exposure, or exposure measured as continuous variable)?  IVs measured as categorical (‘high Agreeableness’) variables upon continuous DV (QoL) | Y |  |  |
| 9. Were the exposure measures (independent variables) clearly defined, valid, reliable, and implemented consistently across all study participants?  Personality traits were assessed using the International Personality Item Pool (IPIP) 50-item Questionnaire (a measure of the Five-Factor Model) and was chosen for this study for its smaller number of items, its reliability evidence, and as its domains show a high correlation to Costa and McCrae’s revised NEO personality inventory (NEO-PI-R).  Participants had received either: vertical sleeve, gastric band, or gastric bypass surgery. Differences in surgery type were not tested or controlled for. | Y |  |  |
| 10. Was the exposure(s) assessed more than once over time?  Cross-sectional research, therefore one time point. |  | N |  |
| 11. Were the outcome measures (dependent variables) clearly defined, valid, reliable, and implemented consistently across all study participants?  *“The Obesity-Related Well-Being Scale (Orwell 97) is a self-reported measure of obesity-related quality of life... In the current study Cronbach’s alpha for the overall Orwell 97 was 0.90, for the symptom occurrence subscale α was 0.72, and for the symptom relevance subscale α was 0.89, indicating that the Orwell 97 is a very reliable assessment of obesity-related well-being.”* | Y |  |  |
| 12. Were the outcome assessors blinded to the exposure status of participants? |  |  | NA |
| 13. Was loss to follow-up after baseline 20% or less? |  |  | NA |
| 14. Were key potential confounding variables measured and adjusted statistically for their impact on the relationship between exposure(s) and outcome(s)?  *“Hierarchical multiple regression was used to assess the predictive value of each of the personality factors for total Orwell 97 scores, symptom occurrence and the relevance of symptoms in separate regressions, after controlling for BMI of respondents.”* | Y |  |  |
| **Determination**  Study measured bariatric operations as one group and justified same. Brief reporting regarding sampling procedures but sufficient detail to determine sample appropriateness. Researchers controlled for important, potentially confounding variables and use of validated scales, as well as sufficient power justification to support results. | **Good** | | |

| **II: Pereira et al. (2019)** | | | |
| --- | --- | --- | --- |
| **Quality Assessment Tool for Before-After (Pre-Post) Studies With No Control Group** | **Y** | **N** | **Other**  **(CD, NR, NA)*** |
| 1. Was the study question or objective clearly stated?  *“This study aimed to assess quality of life in obese patients 1 year after bariatric surgery taking into consideration the influence of socio-demographic, clinical, and psychological variables. …Considering that the literature is not always consensual regarding the role of coping strategies, social support, and impulsiveness in obese patient’s Qol post-surgery, this study focused on QoL 1 year after bariatric surgery.”* | Y |  |  |
| 2. Were eligibility/selection criteria for the study population prespecified and clearly described?  *“The sample included 90 patients submitted to bariatric surgery in a major public hospital in North of Portugal, 1 year after surgery. Participants were 18 or older, with a pre-surgery BMI > 40 kg/m2, and cognitively able to answer questionnaires. Based on medical records, patients with a diagnosis of severe psychiatric disorders were excluded.”* | Y |  |  |
| 3. Were the participants in the study representative of those who would be eligible for the test/service/intervention in the general or clinical population of interest?  *90 patients submitted to bariatric surgery in a major public hospital …. 1 year after surgery. Participants were 18 or older, pre-surgery BMI > 40 kg/m2* | Y |  |  |
| 4. Were all eligible participants that met the prespecified entry criteria enrolled?  *“There were 204 participants that underwent bariatric surgery. However, only 100 were in their first post-surgical year and were, therefore, invited to participate in the study. Only 90 participants were present the day of data collection.”* | Y |  |  |
| 5. Was the sample size sufficiently large to provide confidence in the findings?  Appeared to be sufficient but not reported. N = 90, 9 variables. |  |  | NR |
| 6. Was the test/service/intervention clearly described and delivered consistently across the study population?  *“This study followed a prospective design with two assessment moments. Patients were identified and contacted by the psychologist of the Unit of Psychiatry and Mental Health. Patients were invited by letter and all signed an informed consent. Participation was voluntary. Patients answered the instruments in a room provided by the hospital for that purpose.”* | Y |  |  |
| 7. Were the outcome measures prespecified, clearly defined, valid, reliable, and assessed consistently across all study participants?  *“It is expected that (1) social support, spirituality, and emotion- and problem focused coping strategies will be positively associated with both physical and mental QoL, while eating disorder behavior and impulsiveness will not; (2) spirituality, social support, problem-focused coping, and physical and mental QoL will increase, whereas eating disorder behavior and impulsiveness will not, from the pre-surgery moment to the post-surgery moment; (3) use of more spirituality, less eating disorder behavior, more use of coping strategies lower impulsiveness, and high social support will predict better physical and mental QoL; (4) spirituality will moderate the relationship between impulsiveness and QoL, post-surgery.”*  *“Short Form Health Survey–SF 36 …. assesses QoL through eight subscales Higher scores indicate better perceptions of health status. In the Portuguese validation… these subscales are grouped into two main domains: physical QoL and mental QoL. In this study, the Cronbach alpha for physical QoL was .93 and .95 for mental QoL.”* | Y |  |  |
| 8. Were the people assessing the outcomes blinded to the participants' exposures/interventions?  Due to the study design, blinding likely did not occur, nor would this be considered to impact the research outcomes. |  |  | NR |
| 9. Was the loss to follow-up after baseline 20% or less? Were those lost to follow-up accounted for in the analysis?  (i) 100 eligible, 90 participated in questionnaires. (ii) Unclear, but likely not accounted for in analysis. | Y |  |  |
| 10. Did the statistical methods examine changes in outcome measures from before to after the intervention? Were statistical tests done that provided p values for the pre-to-post changes?  Correlation and regression analyses | Y |  |  |
| 11.* Were key potential confounding variables measured and adjusted statistically for their impact on the relationship between exposure(s) and outcome(s)?  Professional status | Y |  |  |
| 12. If the intervention was conducted at a group level (e.g., a whole hospital, a community, etc.) did the statistical analysis take into account the use of individual-level data to determine effects at the group level? |  |  | NA |
| **Determination:**  Some details omitted, however it was considered these omissions would not heavily impact outcome results. Controlling for further relevant confounding variables may have strengthened study results. | **Good** | | |

| **III: Lee et al. (2011)** | | | |
| --- | --- | --- | --- |
| **Quality Assessment Tool for Before-After (Pre-Post) Studies With No Control Group** | **Y** | **N** | **Other**  **(CD, NR, NA)*** |
| 1. Was the study question or objective clearly stated?  *“This study aims to investigate different clinical outcome variables, the relationships between quality of life and different personality one year after LSG in obese patients.”* | Y |  |  |
| 2. Were eligibility/selection criteria for the study population prespecified and clearly described?  Sample characteristics reported, study did not specify exclusion criteria. However, all participants had available of bariatric surgery, therefore some standards of requirements were applied.  *“This prospective study was conducted in Min-Sheng General Hospital between December 2005 and December 2008. … one year after LSG in obese patients. …Preoperative and one year after laparoscopic surgery evaluation included medical history and physical, nutritional, metabolic cardiopulmonary and psychiatric assessments.”* |  |  | N |
| 3. Were the participants in the study representative of those who would be eligible for the test/service/intervention in the general or clinical population of interest?  *…sample of 61 participants one year after laparoscopic surgery from hospital weigh loss population* | Y |  |  |
| 4. Were all eligible participants that met the prespecified entry criteria enrolled?  Unclear as sample characteristics were reported rather than prespecified eligibility criteria. Unclear whether the same criteria were used for all research participants; and whether all participants who met the criteria enrolled in the study. |  |  | NR |
| 5. Was the sample size sufficiently large to provide confidence in the findings?  This detail was not reported. |  |  | NR |
| 6. Was the test/service/intervention clearly described and delivered consistently across the study population?  LSG for all participants, personality variables measured consistently across participants. | Y |  |  |
| 7. Were the outcome measures prespecified, clearly defined, valid, reliable, and assessed consistently across all study participants?  Outcome variables were clearly defined:  *Clinical variables* (medical history and physical, nutritional, metabolic, cardiopulmonary e.g. systolic blood pressure etc) and *QoL* (measured by Gastrointestinal Quality of Life Index). GQLI is a valid and reliable instrument for measuring QOL, specifically for patients … (Rentz et al., 2001). | Y |  |  |
| 8. Were the people assessing the outcomes blinded to the participants' exposures/interventions?  This detail was not reported. Due to the study design, blinding likely did not occur, nor would this be considered to impact the research outcomes. |  |  | NR |
| 9. Was the loss to follow-up after baseline 20% or less? Were those lost to follow-up accounted for in the analysis?  *“All patients were available for detailed outcome measurements”.* No loss to follow up. | Y |  |  |
| 10. Did the statistical methods examine changes in outcome measures from before to after the intervention? Were statistical tests done that provided p values for the pre-to-post changes?  (i) Yes  (ii) Key statistical tests reported without p values e.g.:  Social functions before LSG “4.8± 0.5”; after LSG “4.9 ± 0.3” |  | N |  |
| 11.* Were key potential confounding variables measured and adjusted statistically for their impact on the relationship between exposure(s) and outcome(s)?  No reported control for potentially confounding variables evidenced within analysis or reporting. |  | N |  |
| 12. If the intervention was conducted at a group level (e.g., a whole hospital, a community, etc.) did the statistical analysis take into account the use of individual-level data to determine effects at the group level? |  |  | NA |
| **Determination**  Methods for sampling appear unclear with regard to eligibility criteria. Unclear how bias was managed in this instance.  Uncertainty regarding the rigour of results: ambiguity re effect sizes as not reported, selective reporting of personality variables (e.g. did not report for ‘industriousness’, ‘other-orientation’, or ‘contentedness’). | **Poor** | | |

| **IV: Canetti et al. (2009)** | | | |
| --- | --- | --- | --- |
| **Quality Assessment Tool for Before-After (Pre-Post) Studies With No Control Group** | **Y** | **N** | **Other**  **(CD, NR, NA)*** |
| 1. Was the study question or objective clearly stated?  *“To examine a structural equation model of the effects of personal* (personality, self-esteem etc.) *and interpersonal factors on treatment outcome* (weight loss, mental health and QoL) *of bariatric surgery and weight loss program”* | Y |  |  |
| 2. Were eligibility/selection criteria for the study population prespecified and clearly described?  *“Surgical patients were recruited among applicants to bariatric surgery at the surgery clinic of Hadassah University Medical Centre. All individuals who were fluent in Hebrew and able to fill out questionnaires were asked to participate in the study. Only one individual refused to participate in the study. Weight loss program individuals were recruited among ….””* | Y |  |  |
| 3. Were the participants in the study representative of those who would be eligible for the test/service/intervention in the general or clinical population of interest?  *“There were two surgery procedures: silastic ring vertical banded gastroplasty (n = 44) and laparoscopic adjustable gastric banding (n = 7). …. The surgical patients were significantly younger, less educated, with lower income, and a younger age of onset of obesity compared with the dieting patients ... They also had a higher proportion of women”* and participants from range of countries*. Surgical participants weighed more, had higher body mass index (BMI), and had lower quality of life at the start of the study, but there were no significant differences in measures of mental health.* | Y |  |  |
| 4. Were all eligible participants that met the prespecified entry criteria enrolled?  *…Only one individual refused to participate in the study. …“All individuals attending the program were asked to participate, 80% agreed to take part in the study. All surgical and dieting participants volunteered to the study. …”* | Y |  |  |
| 5. Was the sample size sufficiently large to provide confidence in the findings?  *The second limitation concerns the number of participants that took part in the study: When the cases/ parameters ratio is less than 10:1 and absolute sample sizes less than 100, the estimates are considered less stable…; therefore our results should be regarded as preliminary.* |  | N |  |
| 6. Was the test/service/intervention clearly described and delivered consistently across the study population?  Surgical group received two different operation types. Not accounted for in analysis. All details clearly described:  *“There were two surgery procedures: silastic ring vertical banded gastroplasty (n 5 44) and laparoscopic adjustable gastric banding (n 5 7). The dieting group participated in a commercial program with medical supervision that combines diet, behavior modification, and physical exercise, with a special emphasis on physical activity.”* | N |  |  |
| 7. Were the outcome measures prespecified, clearly defined, valid, reliable, and assessed consistently across all study participants?  Outcome measures: Weight loss, quality of life, and mental health. Measured the following across sample:  BMI and associated metrics  Medical Outcomes Study Short Form-36 (SF-36)  Mental Health Inventory (MHI) | Y |  |  |
| 8. Were the people assessing the outcomes blinded to the participants' exposures/interventions?  This detail was not reported. |  |  | NR |
| 9. Was the loss to follow-up after baseline 20% or less? Were those lost to follow-up accounted for in the analysis?  **Yes:**  *“No significant differences on initial measures were found between drop outs and the others”.* Dropout calculated at 5%. Surgery T1 = 51, T2 = 44. Full dataset reported in overall demographics. | Y |  |  |
| 10. Did the statistical methods examine changes in outcome measures from before to after the intervention? Were statistical tests done that provided p values for the pre-to-post changes?  **Yes:**  T 1= pre-post op, T 2= 1 yr post op (neuroticism IV of interest, not surgery)  P values reported for relevant tests | Y |  |  |
| 11.* Were key potential confounding variables measured and adjusted statistically for their impact on the relationship between exposure(s) and outcome(s)?  Correlation analysis:  *“As the initial levels of the outcome variables were strongly correlated with improvement, partial correlation coefficients were calculated between predictor and outcome variables, controlling for the initial level of the outcome variable.”* | Y |  |  |
| 12. If the intervention was conducted at a group level (e.g., a whole hospital, a community, etc.) did the statistical analysis take into account the use of individual-level data to determine effects at the group level?  Not applicable |  |  | NA |
| **Determination:**  Differences in surgical techniques were not accounted for in analysis. However, evidence debates whether differences in QoL outcomes are due to surgery type therefore, may not impact results. Eligibility criteria specified. Appropriate management of covariates applied in analysis, however effects of weight loss (not controlled for) suspected to have impacted results. Underpowered sample size exposed the results to risk of bias, potentially impacting outcome results. | **Fair** | | |

| **V: van Hout et al. (2009)** | | | |
| --- | --- | --- | --- |
| **Quality Assessment Tool for Before-After (Pre-Post) Studies With No Control Group** | **Y** | **N** | **Other**  **(CD, NR, NA)*** |
| 1. Was the study question or objective clearly stated?  *“The present study investigated in morbid obese patients who underwent vertical banded gastroplasty (VBG) the predictive value of preoperative parameters, especially health-related quality of life (HRQoL), personality, psychosocial functioning, body image, and eating behavior for 2- year changes in these variables, as well as weight loss.”* | Y |  |  |
| 2. Were eligibility/selection criteria for the study population prespecified and clearly described?  *“Preoperatively, 146 patients who underwent VBG between January 2001 and February 2005 were psychologically assessed. In addition to the International Federation for the Surgery of Obesity inclusion criteria…], 10% preoperative weight loss was required.”* | Y |  |  |
| 3. Were the participants in the study representative of those who would be eligible for the test/service/intervention in the general or clinical population of interest?  *…“patients who underwent VBG between January 2001 and February 2005. 98 (87.5%) female and 14 (12.5%) male patients…. Preoperatively, they had a mean age of 38.8± 8.3 years, an average body mass index (BMI) of 45.3±5.1 and a mean percentage excess weight (EW) of 104.2± 22.4%.”* | Y |  |  |
| 4. Were all eligible participants that met the prespecified entry criteria enrolled?  *“Preoperatively, 146 patients who underwent VBG between January 2001 and February 2005 were psychologically assessed. In addition to the International Federation for the Surgery of Obesity inclusion criteria…, 10% preoperative weight loss was required. From these 146 patients, 112 (76.7%) could be included; despite repeated requests, other patients did not return the test booklet.”* | Y |  |  |
| 5. Was the sample size sufficiently large to provide confidence in the findings?  *…“sample size guidelines of Tabachnick and Fidell…, a maximum of eight independent psychosocial predictors were entered in the analyses.”* | Y |  |  |
| 6. Was the test/service/intervention clearly described and delivered consistently across the study population?  The study investigated patients who each underwent vertical banded gastroplasty. Measures of personality appeared to be applied throughout the sample. | Y |  |  |
| 7. Were the outcome measures prespecified, clearly defined, valid, reliable, and assessed consistently across all study participants?  Preoperatively as well as 2 years after surgery, all candidates completed a semi-structured interview and a battery of questionnaires” . Only questionnaire data reported in the study.  Outcome variables: HR-QoL, personality, psychosocial functioning, body image, eating behaviour, and weight loss:  Dutch version of the Rand-36 (HR-QoL)  Dutch Personality Questionnaire (personality: neuroticism and social anxiety)  Amsterdam Biographical Questionnaire (personality: neurotic lability-somatic symptoms) | Y |  |  |
| 8. Were the people assessing the outcomes blinded to the participants' exposures/interventions?  This detail was not reported due to the study design. |  |  | NA |
| 9. Was the loss to follow-up after baseline 20% or less? Were those lost to follow-up accounted for in the analysis?  *“From these 146 patients, 112 (76.7%) could be included”*  Loss to follow up = >20%  Those lost to follow up not accounted for. |  | N |  |
| 10. Did the statistical methods examine changes in outcome measures from before to after the intervention? Were statistical tests done that provided p values for the pre-to-post changes?  **Yes**  *“Preoperatively as well as 2 years after surgery, all candidates completed a semi-structured interview and a battery of psychological questionnaires”.*  Results reported as *“Prediction of Change 2 Years After Surgery”.* Also adjusted to more stringent significance level (p≤ 0.01) because of the multiple tests. | Y |  |  |
| 11.* Were key potential confounding variables measured and adjusted statistically for their impact on the relationship between exposure(s) and outcome(s)?  Age, gender, preoperative BMI | Y |  |  |
| 12. If the intervention was conducted at a group level (e.g., a whole hospital, a community, etc.) did the statistical analysis take into account the use of individual-level data to determine effects at the group level?  Intervention was not conducted at a group level (e.g. not whole community). |  | NA |  |
| **Determination**  Relevant control variables included applied, justification re sample power, appropriate recruitment and testing procedures. | **Good** | | |
