# Supplementary material for: Personality trait associations with quality-of-life outcomes following bariatric surgery: a systematic review
Source: Health Qual Life Outcomes. 2023 Mar 29;21:32. doi: 10.1186/s12955-023-02114-0 (PMC10061792; doi:10.1186/s12955-023-02114-0)
Supplement: Supplementary file 5 — Additional file 5: Table 5. Quality assessment for observational cohort and cross-sectional studies. Table 6. Quality assessment for pre/post studies with no control. [file 12955_2023_2114_MOESM5_ESM.docx]

**Additional file 5.**

**Table 5.**

*Quality assessment for observational cohort and cross-sectional studies*

| **Author** | **Q1** | **Q2** | **Q3** | **Q4** | **Q5** | **Q6** | **Q7** | **Q8** | **Q9** | **Q10** | **Q11** | **Q12** | **Q13** | **Q14** | **Rating** | **Rater 1** | **Rater 2** |
| --- | --- | --- | --- | --- | --- | --- | --- | --- | --- | --- | --- | --- | --- | --- | --- | --- | --- |
|  |  |  |  |  |  |  |  |  |  |  |  |  |  |  |  |  |  |
| (Caltabiano, 2021) | Y | Y | CD | Y | Y | N | NA | Y | Y | N | Y | NA | NA | Y | Good | SS | EK |
|  |  |  |  |  |  |  |  |  |  |  |  |  |  |  |  |  |  |

**Table 6.**

*Quality assessment for pre/post studies with no control*

| **Author** | **Q1** | **Q2** | **Q3** | **Q4** | **Q5** | **Q6** | **Q7** | **Q8** | **Q9** | **Q10** | ***Q11** | **Q12** | **Rating** | **Rater 1** | **Rater**  **2** |
| --- | --- | --- | --- | --- | --- | --- | --- | --- | --- | --- | --- | --- | --- | --- | --- |
|  |  |  |  |  |  |  |  |  |  |  |  |  |  |  |  |
| Pereira et al. (2019) | Y | Y | Y | Y | NR | Y | Y | NR | Y | Y | Y | NA | Good | SS | - |
|  |  |  |  |  |  |  |  |  |  |  |  |  |  |  |  |
| Lee et al. (2011) | Y | N | Y | NR | NR | Y | Y | NR | Y | N | N | NA | Poor | SS | - |
|  |  |  |  |  |  |  |  |  |  |  |  |  |  |  |  |
| Canetti et al. (2009) | Y | Y | Y | Y | N | N | Y | NR | Y | Y | Y | NA | Fair | SS | EK |
|  |  |  |  |  |  |  |  |  |  |  |  |  |  |  |  |
| Van Hout et al. (2009) | Y | Y | Y | Y | Y | Y | Y | NA | N | Y | Y | NA | Good | SS | EK |
|  |  |  |  |  |  |  |  |  |  |  |  |  |  |  |  |

*Replaced the original Q 11 (*“Were outcome measures of interest taken multiple times before the intervention and multiple times after the intervention?”*) as this was not relevant for the studies presented, with Q14 from ‘Observational / cross sectional’ to assess for impact of confounding variables (“*Were key potential confounding variables measures and adjusted statistically for their impact on the relationship between exposure(s) and outcome(s)?”.*
